# Task-aware conformal power envelopes for multi-horizon spacecraft monitoring

**DOI:** 10.1371/journal.pone.0360910

**Published:** 2026-10-13

**Authors:** Nan Lu, Lehan Cui, Xinian Zhi

**Affiliations:** 1 School of Computer Science, Northwestern Polytechnical University, Xi’an, China; 2 Ningbo Institute, Northwestern Polytechnical University, Ningbo, China; Swiss Federal Technology Institute of Lausanne, SWITZERLAND

## Abstract

Reliable spacecraft power monitoring requires uncertainty estimates across forecast horizons and consistency between primitive electrical states and derived power. We present a task-aware conformal framework that selects joint residual geometry according to downstream power-envelope efficiency. A residual iTransformer produced point centres for five primitive electrical states at 1, 5, and 15 min. Candidate geometries were fitted, selected, and calibrated on chronologically separated complete-orbit blocks, and the final evaluation comprised 25,278 forecast origins from the chronologically subsequent July 2023 telemetry. Across three random-seed runs, the task-aware selector chose a joint box, whereas a residual-volume control selected the sample-covariance ellipsoid. Orbit-balanced primitive-state joint coverage was 0.892 (95% complete-orbit bootstrap confidence interval [CI], 0.883–0.899) at the 90% nominal level and 0.947 (95% CI, 0.943–0.952) at the 95% nominal level. At 90% and 95%, simultaneous coverage of both power channels across all horizons was 0.913 and 0.957. Relative to residual-volume selection, the task-aware method reduced normalized power interval score by 34.3% and 30.3%; the paired absolute reductions were 1.522 (95% CI, 1.505–1.540) and 1.552 (95% CI, 1.534–1.570). Power envelopes were computed from synchronized primitive current and bus-voltage sets. For the selected non-negative box, zero algebraic gap and full interval-image containment follow by construction; the empirical analyses quantify coverage and sharpness. The results demonstrate task-aware geometry selection for BUPT-1 multi-horizon power monitoring.

## Introduction

Electrical power is a mission-limiting resource for small satellites and onboard computing payloads. Current, voltage, charge, load, illumination, and thermal conditions evolve together over an orbit, and the power quantities used in monitoring are products of correlated primitive measurements. Public BUPT-1 measurements show that power availability and payload demand vary materially across orbital conditions [1]. Recent satellite power research has therefore considered probabilistic segmentation [2] and online state prediction under changing operating conditions [3].

Spacecraft telemetry forecasting has also combined global and local machine-learning components to represent trend and irregular variation while providing point and interval outputs [4]. The wider telemetry literature emphasizes heterogeneous channels, temporal dependence, limited labelled events, and the need for operationally interpretable monitoring outputs [5]. These studies motivate uncertainty estimates that are both multivariate and compatible with the physical quantities consumed by power-system monitoring.

Modern time-series architectures improve point forecasts by representing cross-variable dependence, periodicity, and multiscale structure. iTransformer learns attention across variables [6], CycleNet models recurrent cycles [7], and TimeMixer separates temporal information across scales [8]. These methods strengthen forecast centres, but a point estimate alone does not state which combinations of future currents and voltages are jointly plausible.

Conformal prediction supplies model-agnostic uncertainty sets without prescribing a parametric noise distribution [9]. Beyond time-series forecasting, conformal prediction has also been applied to uncertainty quantification in dynamic biological systems [10]. Extensions for distribution shift, non-exchangeable observations, multivariate time series, and multi-horizon output have appeared rapidly [11–16]. Ellipsoidal regions retain residual dependence [13], while optimized convex templates demonstrate that region shape affects efficiency [17,18]. In spacecraft energy monitoring, however, the final quantities of interest include derived total and solar power. A region that is compact in residual space need not produce the sharpest power envelope after bilinear propagation.

We formulate uncertainty geometry as a downstream task-selection problem. Candidate box and covariance-based families are fitted on one complete-orbit block; a second block selects the geometry that minimizes normalized total- and solar-power interval width; and a third block calibrates the selected score. A residual-volume selector, which does not use power, is included as a control. The selected primitive-state set is intersected with the observed non-negative physical domain and propagated through synchronized electrical equations. Power envelopes are obtained exclusively from the calibrated primitive-state set, without a separate power-calibration stage.

The study makes three contributions. First, it isolates downstream task-aware selection from residual-space compactness by adding a non-task-aware geometry-selection control. Second, it evaluates power-channel coverage directly at each horizon and across all six power outcomes, with complete-orbit bootstrap confidence intervals and multiplicity-adjusted paired comparisons. Third, it distinguishes the structural algebraic consistency of interval propagation from the empirical coverage and sharpness results. These contributions are evaluated on the public BUPT-1 benchmark using three random-seed runs of a common point-model architecture.

## Materials and methods

### Data source, preprocessing, and electrical variables

The source data were released with the SatelliteCOTS measurement study [1]. We used the public CommonData-Telemetries record from the BUPT-1 12U CubeSat and pinned the source checkout to commit 951b41521351d535b7c2354916d9c4991602e8c7. The original telemetry contains 10,117,299 rows. Timestamps were parsed and sorted, samples were grouped by floored 10-s timestamps, and arithmetic means were computed within each bin. Bins with fewer than eight raw samples were removed; a second duplicate-timestamp check after chunk merging retained the first duplicate, although the final table contained no duplicate 10-s timestamps. The resulting 1,011,745 bins contained no missing values in the audited electrical channels.

A discontinuity was declared whenever two retained bin timestamps differed by other than 10 s; the first row also began a segment. This produced 35 continuous source segments. A forecast origin was retained only when its 600-bin input history and all targets remained within one segment. The 13 input channels were total current, bus voltage, two maximum-power-point-tracker (MPPT) output currents, total power, solar power, battery voltage, battery current, communication power, payload power, a sunlit indicator, and sine and cosine of a deterministic 90-min orbit-phase feature. Training-set means and population standard deviations standardized all continuous features; no July statistic entered scaling.

The five primitive targets were total current (A), bus voltage (V), MPPT1 output current (A), MPPT2 output current (A), and battery voltage (V), each at 1, 5, and 15 min. Total power was total current multiplied by bus voltage. Solar power was the sum of the two MPPT output currents multiplied by the same bus-voltage channel. All current and voltage terms were arithmetic means from the same retained 10-s bin before power was derived. Across training, June development, and July evaluation, the five primitive variables had zero missing and zero negative values; period-specific ranges are reported in S2 Table.

Table 1 reports the chronological allocation. March 22–May 31 was used to fit point-model parameters. Point-model early stopping monitored June 1–30 validation loss; after the checkpoint was fixed, June residuals were divided into complete-orbit blocks for geometry fitting, geometry selection, and calibration. July 1–24 formed the final evaluation set. The 90% and 95% levels are stated as nominal calibration levels; July empirical coverage and complete-orbit confidence intervals quantify achieved performance under the chronological protocol.

**Table 1:** Chronological data allocation.

Role	Calendar interval	Retained 10-s bins	Continuous segments	Forecast origins
Model training	2023-03-22–2023-05-31	603549	25	98158
Point-model early stopping; geometry and conformal development	2023-06-01–2023-06-30	253089	5	41610
Final evaluation	2023-07-01–2023-07-24	155107	7	25278

Calendar intervals are disjoint. Split-specific segment counts may share a source segment at a calendar boundary. Forecast-origin counts apply the common history, horizon, and stride rules.

### Point-forecast centres

The forecast centre combined a closed-form ridge autoregressive prior with a compact residual iTransformer. The ridge component captured local linear persistence, while the residual encoder learned cross-variable corrections. The model produced the five primitive states at all three horizons in one forward pass:


$$\begin{array}{c} ({\hat{y}}_{t+h}\;=\;f_{\theta }\left(x_{t-L+\text{1}:t}\right),\text{ h }\in \;\{\text{1},\;\text{5},\text{ 15}\}\text{ min} \end{array}$$ (1)

Ridge autoregression, CycleNet [7], iTransformer [6], and the residual iTransformer were evaluated under the same chronological split, 100-min context, and target scaling. Neural-model results use random seeds 7, 42, and 101; these are three runs of the same architecture, not three model classes. Table 2 reports the configurations and parameter counts. Architecture, hyperparameter, and checkpoint selection were completed using the June development period before July evaluation.

**Table 2:** Point-model and training configurations.

Model	Core specification	Context	Trainable parameters	Training
Ridge autoregression	Direct linear autoregression; regularization selected on validation data	100 min	deterministic fit	closed-form ridge; candidate regularization 0.01–100
CycleNet	540-bin learnable cycle and linear residual projection	100 min	4,503	AdamW, lr 1e-3, batch 256, up to 20 epochs
iTransformer	64-dimensional variate tokens; 4 heads; 2 encoder layers	100 min	105,731	AdamW, lr 5e-4, batch 128, up to 15 epochs
Residual iTransformer (point centre)	Ridge prior plus 64-dimensional, 4-head, 2-layer residual encoder	100 min	159,823	AdamW, lr 5e-4, batch 128, up to 15 epochs

RMSE comparisons use the same 25,278 July forecast origins. Neural entries are run for random seeds 7, 42, and 101; ridge regression is deterministic.

The study design, residual-volume control, and algebraic propagation are summarized in Fig 1.

**Fig 1:**
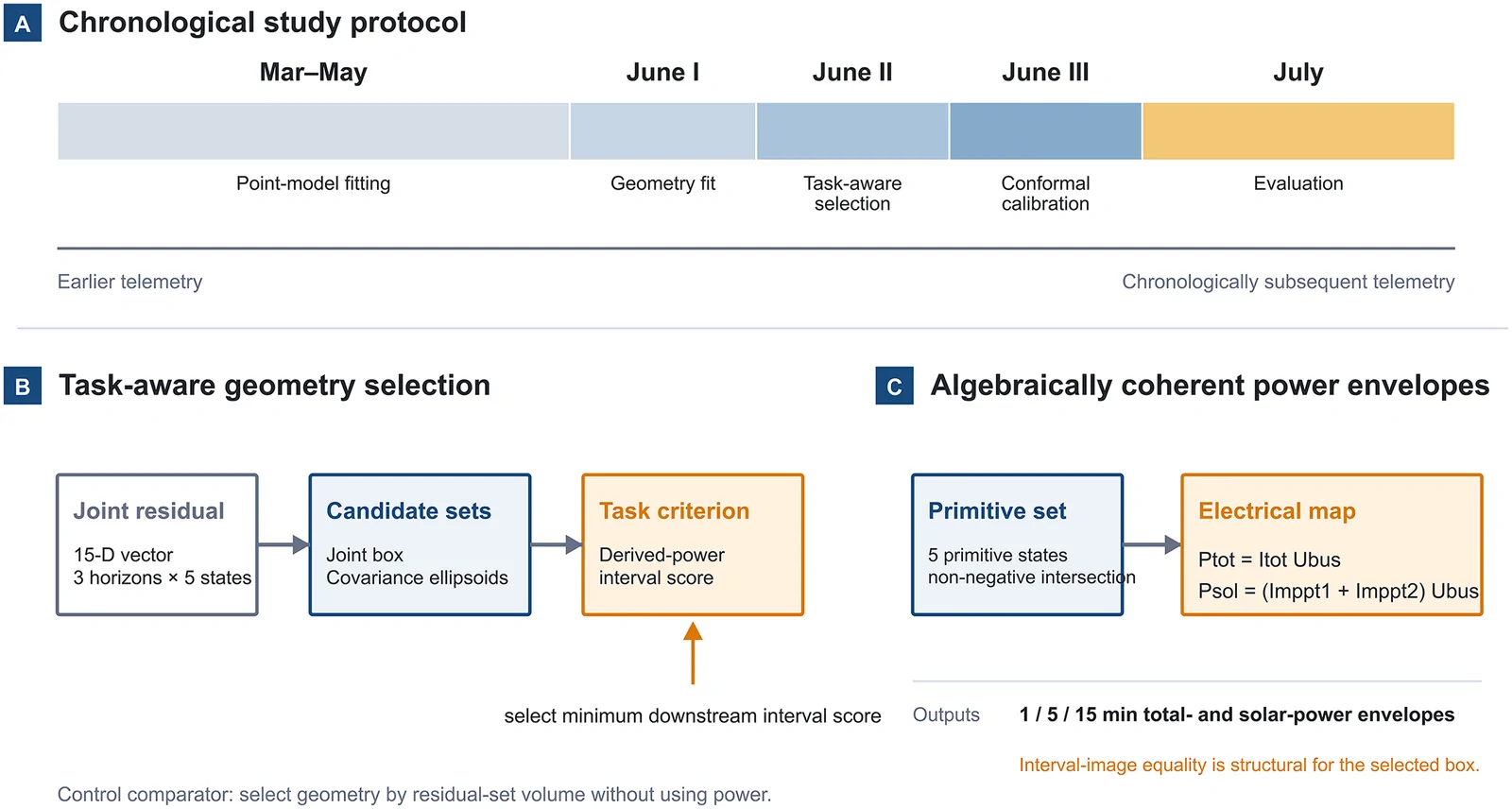
Chronological protocol and task-aware uncertainty construction. (A) Point-model fitting, geometry fitting, task-aware selection, conformal calibration, and evaluation are ordered in time. (B) Candidate residual geometries are compared using the downstream power criterion; a residual-volume selector is the non-task-aware control. (C) synchronized primitive-state sets are propagated through the total- and solar-power equations. The selected-box interval-image equality is structural.

The calendar allocation and electrical activity over the three periods are shown in Fig 2.

**Fig 2:**
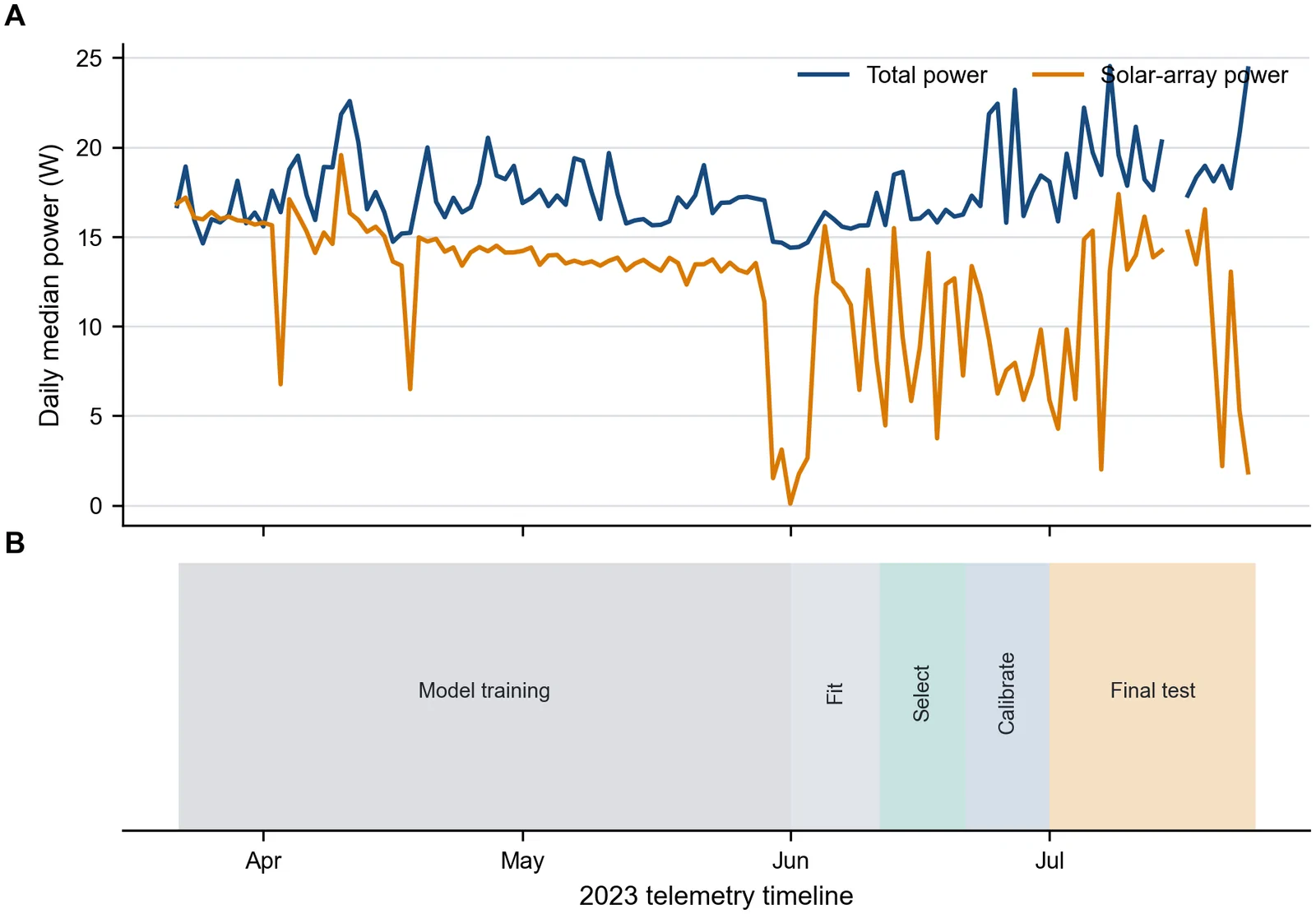
Chronological data protocol. Daily median total and solar power are shown above the model-training, June development, and July evaluation periods. Complete June orbits are divided chronologically into geometry-fit, geometry-selection, and conformal-calibration thirds.

### Primitive and derived outputs

The uncertainty layer was calibrated on 15 primitive standardized residuals: five variables at three horizons. Derived total and solar power were computed as


$$\begin{array}{c} P_{tot}\;=\;I_{tot}U_{bus},\;P_{sol}\;=\;\left(I_{mppt\text{1}}\;+\;I_{mppt\text{2}}\right)U_{bus} \end{array}$$ (2)

Both power definitions use synchronized current and bus-voltage channels from the same retained 10-s bin, consistent with the preprocessing pipeline and stored power targets. Measurement units, observed ranges, and negative fractions are reported in S2 Table.

All audited primitive targets were non-negative, so each candidate set was intersected with the non-negative orthant before power propagation. Omitting this intersection left primitive joint coverage unchanged across every seed and nominal level and produced wider power intervals and higher interval scores. This sensitivity result supports the non-negative-domain intersection for the observed operating range.

### Leakage-controlled geometry selection

Geometry development used 467 complete June orbits. The first chronological third (155 orbits) estimated score geometry, the second (156 orbits) selected the family, and the final third (156 orbits) calibrated the conformal radius. Candidate families comprised a standardized axis-aligned joint box, sample-covariance and diagonal ellipsoids, oracle-approximating-shrinkage (OAS) covariance [19], and covariance-shrinkage ellipsoids with λ∈{0,0.25,0.50,0.75,1}. OAS denotes oracle approximating shrinkage.

For a residual vector e and positive coordinate scales d, the box score was


$$\begin{array}{c} s_{box}(e)\;=\;{\text{max}}_{j}\;\frac{\left|e_{j}\right|}{d_{j}} \end{array}$$ (3)

The ellipsoidal score used


sell(e; λ) = eTΣλ−1e,    Σλ = (1−λ)Σ^ + λ diag(Σ^) (4)

Each candidate was provisionally calibrated within the geometry-fit block. The task-aware selector minimized an equally weighted average of normalized interval widths over two power channels and three horizons on the independent selection block:


$$\begin{array}{c} g^{*}\;=\;{\text{arg min}}_{g\in G}\;\frac{\text{1}}{N_{sel}}\;\sum_{i}\;{\text{Width}}_{P}\left(C_{g,\alpha }\left(x_{i}\right)\right) \end{array}$$ (5)

For each power channel and horizon, physical interval width was divided by that power target’s training-set standard deviation; all six normalized widths then received weight 1/6. As a non-task-aware control, residual-volume selection minimized log volume in the 15-dimensional standardized primitive-residual space. For boxes, log volume was the sum of log side lengths; for ellipsoids, it was the log determinant term plus the calibrated-radius term, with constants common to all candidates omitted. Selection was repeated for each nominal level and random seed. Alternative 25/40%, 33/33%, and 40/35% fit/selection allocations selected the joint box in all nine seed-partition combinations (S4 Table).

### Conformal calibration and completed-orbit update

After the family had been selected, scores were recomputed on the independent final June block. With n calibration scores sorted in ascending order, the split-conformal radius was the order statistic


$$\begin{array}{c} q_{\text{1}-\alpha }\;=\;s_{\lceil (n+\text{1})(\text{1}-\alpha )\rceil } \end{array}$$ (6)

During July evaluation, the radius was updated only after a complete orbit, using a trailing window of 112 complete orbits (approximately seven days). The 112-orbit history was fixed before examining July results as a one-week operational memory. Scores from the active orbit were unavailable and could not influence that orbit’s predictions. Sensitivity analyses used 56 and 224 orbits and a static June radius; coverage and interval score remained close across these histories (S1 Table).


$$\begin{array}{c} C_{t,\alpha }\;=\;\{y\;:\;s_{g^{*}}\left(y\;-\;{\hat{y}}_{t}\right)\;\leq \;q_{\text{1}-\alpha ,t}\}\;\cap \;{R_{\geq \text{0}}}^{\text{1}\text{5}} \end{array}$$ (7)

For the selected box, interval arithmetic gives the exact image of each bilinear power expression over the non-negative primitive box. Zero algebraic gap and full containment are therefore by-construction properties, while empirical coverage and interval score are data-dependent results. For ellipsoidal baselines, power extrema were computed numerically from the corresponding primitive region; a direct baseline calibrated a 21-dimensional ellipsoid after appending six derived-power targets.

### Evaluation and statistical analysis

Point forecasts were summarized by macro standardized root-mean-square error (RMSE) across the five primitive targets. For each channel and horizon, physical root-mean-square error was divided by the corresponding training-set standard deviation; the five standardized errors were averaged with equal weights. Uncertainty methods were summarized by sample-weighted coverage, orbit-balanced coverage, normalized width, and normalized interval score. Orbit-balanced metrics first average within each complete July orbit and then assign equal weight to each orbit.

For a power interval [l,u], observation y, and target miscoverage α, interval score [20] was


$$\begin{array}{c} {IS}_{\alpha }(l,u;y)\;=\;(u-l)\;+\;\frac{\text{2}}{\alpha }(l-y)\text{1}\left\{y<l\right\}\;+\;\frac{\text{2}}{\alpha }(y-u)\text{1}\left\{y>u\right\} \end{array}$$ (8)

The score was computed in physical units, divided by the corresponding training-set power standard deviation, and averaged equally over total power, solar power, and the three horizons. Reported normalized scores are dimensionless. Primitive-state joint coverage requires all 15 coordinates to be covered; power-channel joint coverage requires both power outcomes at a given horizon; all-power joint coverage requires all six power outcomes. Marginal coverage is reported for every primitive coordinate and power channel.

Uncertainty was estimated with 2,000 paired bootstrap replicates that resampled complete July orbits and preserved method pairing. We report percentile 95% confidence intervals for the task-aware estimates and paired score differences. Eight planned comparator-by-target tests were evaluated with two-sided paired orbit-bootstrap P values and Holm adjustment. Random seeds were averaged within each orbit before paired resampling, so inferential intervals reflect orbit-to-orbit variation rather than treating seeds or overlapping forecast origins as independent replicates.

## Results

### Point-centre forecasting

The residual iTransformer had the lowest mean macro standardized RMSE at all three horizons (Table 3): 0.229, 0.425, and 0.577 at 1, 5, and 15 min. It therefore supplied the common point centre for all uncertainty comparisons; uncertainty methods differed only in residual-set construction. Fig 3 summarizes these point-forecast results across horizons.

**Table 3:** July point-forecast macro standardized RMSE.

Model	1 min	5 min	15 min
Ridge autoregression	0.243	0.470	0.638
CycleNet	0.253 ± 0.002	0.475 ± 0.001	0.641 ± 0.001
iTransformer	0.247 ± 0.003	0.427 ± 0.005	0.592 ± 0.012
Residual iTransformer (point centre)	0.229 ± 0.001	0.425 ± 0.008	0.577 ± 0.004

Values are dimensionless. Neural entries are mean ± standard deviation across three random seeds; lower is better.

**Fig 3:**
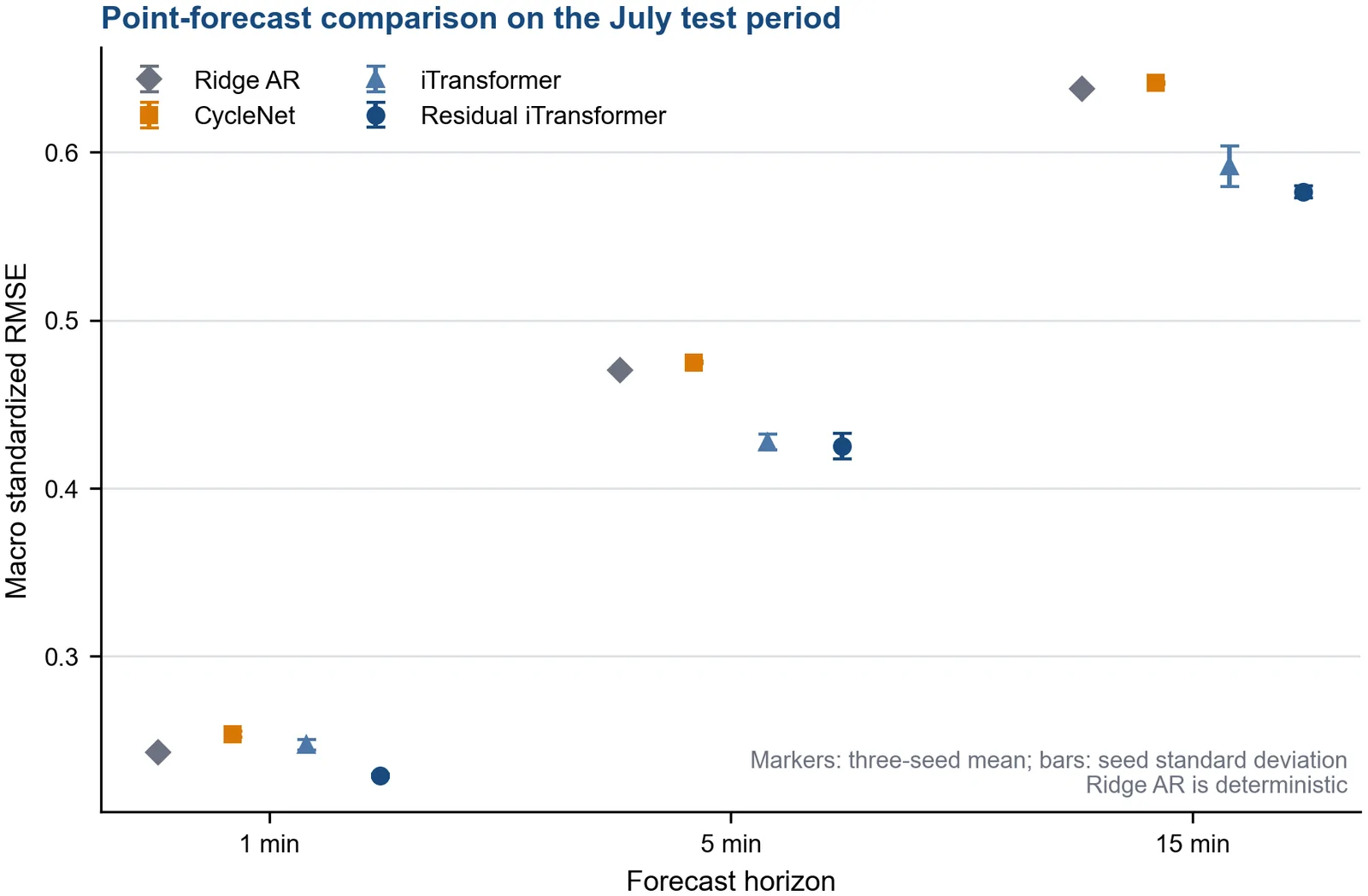
Point-forecast performance on 25,278 July origins. Bars show macro standardized RMSE across five primitive targets; neural error bars denote one standard deviation across three random seeds.

### Geometry selection and non-task-aware control

The task-aware selector chose the joint box in every seed at both nominal levels (Table 4). In contrast, the residual-volume control chose the sample-covariance ellipsoid (shrinkage λ = 0) in all three seeds. This reversal isolates the role of the downstream power objective: the geometry compact in standardized residual space was not the geometry with the best induced power score. The selected-box result was unchanged in all nine alternate chronological orbit-block assignments (S4 Table). Fig 4 compares the candidate geometries on the independent June selection block.

**Table 4:** Geometry-selection power width on the independent June selection block.

Geometry	90%	95%
Joint box	3.060 ± 0.043	3.661 ± 0.048
Ellipsoid, shrinkage 0	4.689 ± 0.051	5.263 ± 0.077
Ellipsoid, shrinkage 0.25	3.950 ± 0.038	4.547 ± 0.031
Ellipsoid, shrinkage 0.50	3.916 ± 0.033	4.578 ± 0.042
Ellipsoid, shrinkage 0.75	4.066 ± 0.026	4.784 ± 0.045
Ellipsoid, shrinkage 1.00	4.516 ± 0.011	5.329 ± 0.068
OAS ellipsoid	4.596 ± 0.045	5.165 ± 0.072

Entries are mean ± standard deviation across three random seeds. Width is normalized by training-set power scales and averaged equally over two power channels and three horizons; lower is better.

**Fig 4:**
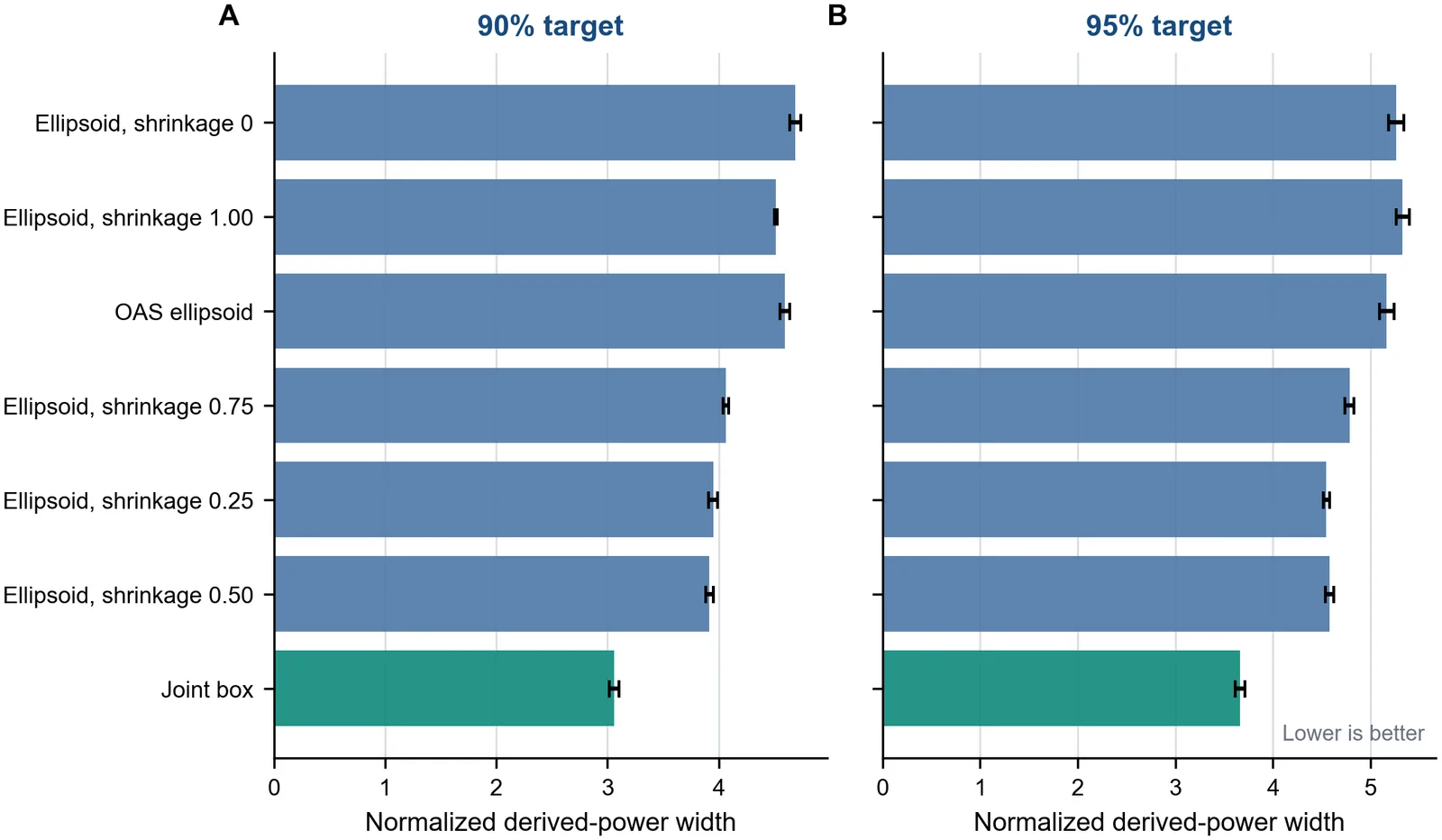
Candidate-geometry selection on the independent June orbit block. Points show normalized total- and solar-power envelope width; lower values indicate sharper envelopes. Error bars denote one standard deviation across three random seeds.

### Uncertainty-set performance

Table 5 summarizes the full method comparison. The task-aware method had the lowest normalized power interval score at both nominal levels while preserving primitive-state coverage near the target. Table 5 reports sample-weighted seed summaries; Fig 5 reports orbit-balanced estimates with 95% complete-orbit bootstrap confidence intervals and includes the residual-volume control.

**Table 5:** July uncertainty-set evaluation.

Method	Target	Joint coverage	Power width	Power interval score	Algebraic gap	Envelope containment
Direct 21-D ellipsoid	90%	0.890 ± 0.000	5.404 ± 0.041	5.412 ± 0.040	0.0659	0.0036
Ellipsoid-only selection	90%	0.891 ± 0.001	3.585 ± 0.041	3.651 ± 0.043	not applicable	not applicable
OAS ellipsoid	90%	0.893 ± 0.001	4.325 ± 0.032	4.351 ± 0.032	not applicable	not applicable
Task-selected geometry	90%	0.892 ± 0.001	2.754 ± 0.044	2.918 ± 0.048	0.0000	1.0000
Task-selected geometry, no orbit update	90%	0.875 ± 0.001	2.609 ± 0.034	2.803 ± 0.039	0.0000	1.0000
Direct 21-D ellipsoid	95%	0.944 ± 0.000	6.266 ± 0.059	6.272 ± 0.058	0.0691	0.0024
Ellipsoid-only selection	95%	0.946 ± 0.000	4.303 ± 0.070	4.359 ± 0.070	not applicable	not applicable
OAS ellipsoid	95%	0.947 ± 0.000	5.006 ± 0.050	5.031 ± 0.049	not applicable	not applicable
Task-selected geometry	95%	0.947 ± 0.000	3.424 ± 0.068	3.573 ± 0.069	0.0000	1.0000
Task-selected geometry, no orbit update	95%	0.935 ± 0.003	3.242 ± 0.032	3.425 ± 0.043	0.0000	1.0000

Values are sample-weighted mean ± standard deviation across three random seeds. Joint coverage refers to all five primitive targets and all three horizons. Width and interval score are dimensionless; lower is better. ‘Not applicable’ denotes ellipsoid projections that do not define coordinate intervals for an interval-image audit.

**Fig 5:**
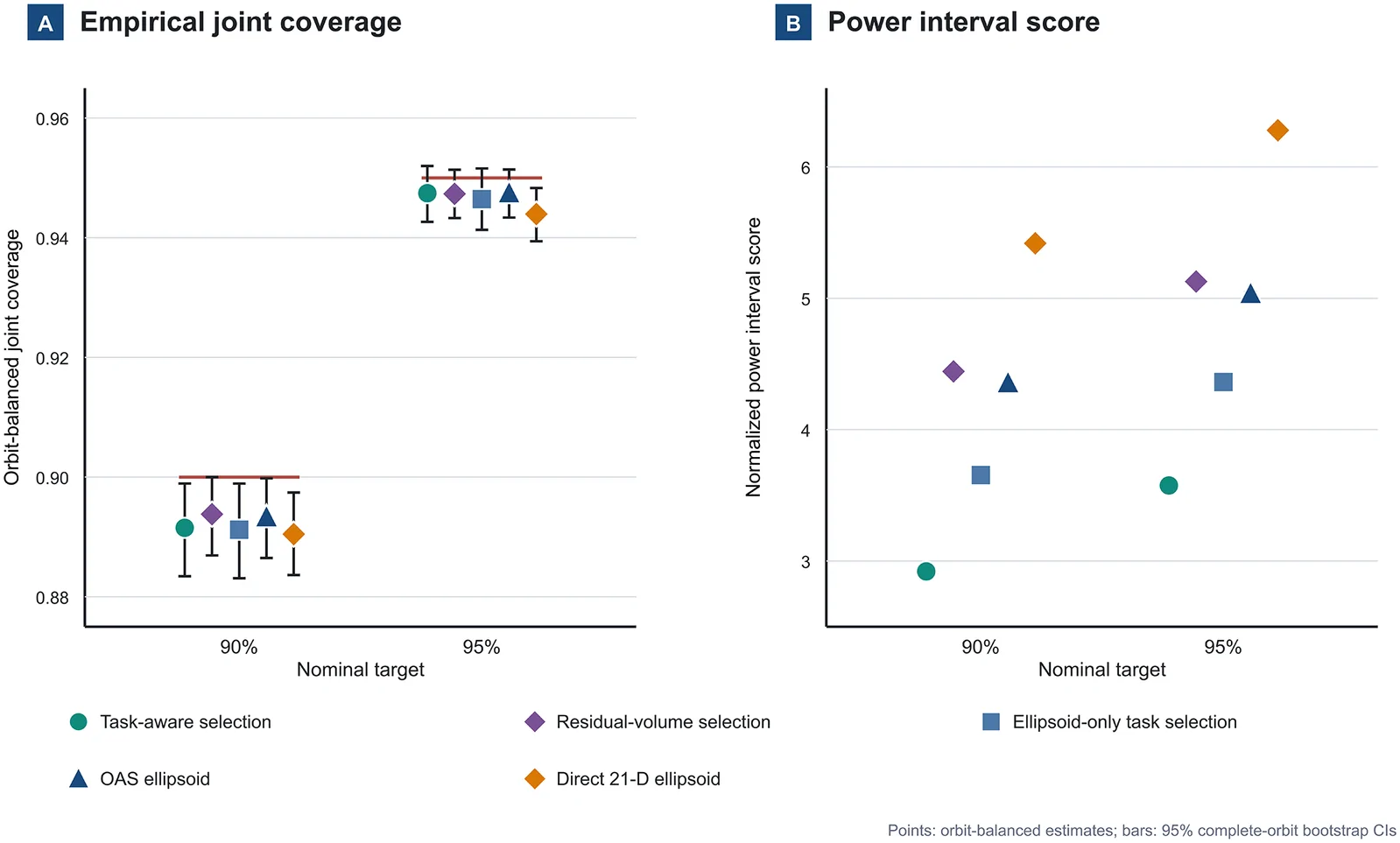
Primitive-state joint coverage and power-envelope efficiency. Points are orbit-balanced estimates and bars are 95% paired complete-orbit bootstrap confidence intervals. Red horizontal marks show nominal coverage. Each estimate averages three random seeds; n = 25,278 July origins per seed.

### Representative orbit envelope

A complete orbit was selected by the pre-specified median 15-min point-error rule. Its total- and solar-power trajectories are displayed with the selected non-negative envelopes in Fig 6.

**Fig 6:**
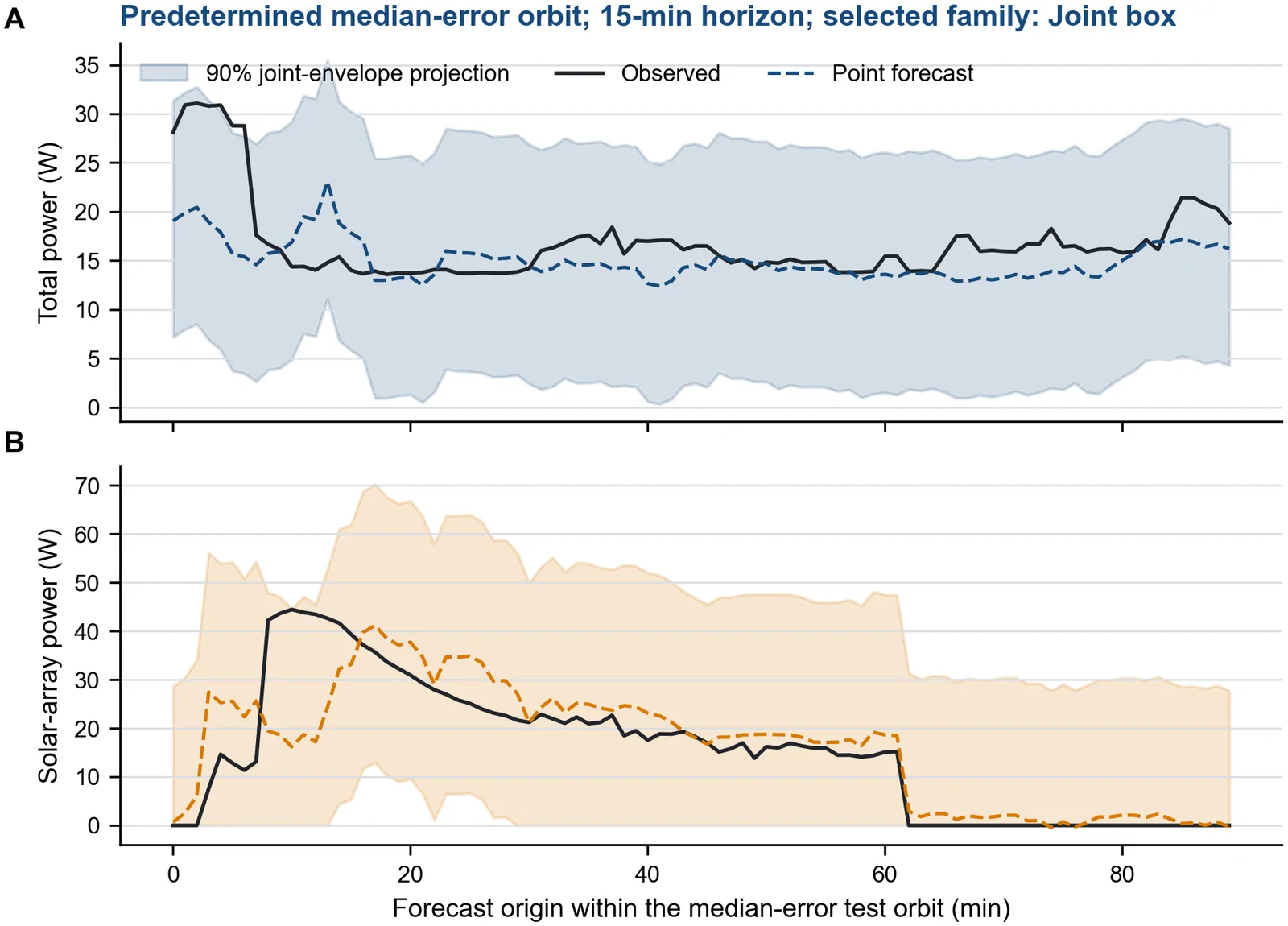
Representative 15-min spacecraft power envelopes. The orbit was selected by median point error using a pre-specified rule. Shading shows completed-orbit calibrated envelopes; solid lines show observed power. Power is derived from synchronized primitive current and bus-voltage channels.

### Primitive and power coverage

At the 90% and 95% nominal calibration levels, the task-aware method attained orbit-balanced July primitive-state joint coverage of 0.892 (95% CI, 0.883–0.899) and 0.947 (95% CI, 0.943–0.952), respectively. Sample-weighted estimates were 0.893 and 0.947.

Coverage of the operational power quantities was higher than primitive 15-dimensional joint coverage at individual horizons (Table 6; Fig 7). At the 90% nominal level, total-power marginal coverage was 0.980–0.991 across horizons, solar-power marginal coverage was 0.975–0.984, and simultaneous coverage of both channels was 0.966–0.968. Across all horizons jointly, coverage was 0.965 for total power, 0.944 for solar power, and 0.913 for all six power outcomes. Corresponding 95% nominal results were 0.984, 0.972, and 0.957. Primitive coordinate-by-horizon coverage is reported in S3 Table.

**Table 6:** July empirical coverage of the two power channels.

Target	Power channel	1 min	5 min	15 min	All horizons jointly
90%	Total power	0.980	0.990	0.991	0.965
90%	Solar power	0.984	0.977	0.975	0.944
90%	Both power channels jointly	0.966	0.968	0.967	0.913
95%	Total power	0.991	0.995	0.996	0.984
95%	Solar power	0.991	0.988	0.991	0.972
95%	Both power channels jointly	0.982	0.983	0.988	0.957

Values average three random seeds over 25,278 forecast origins per seed. The first two rows at each target are marginal by channel; ‘both power channels jointly’ requires simultaneous coverage of both channels. The final column requires the stated event at all three horizons.

**Fig 7:**
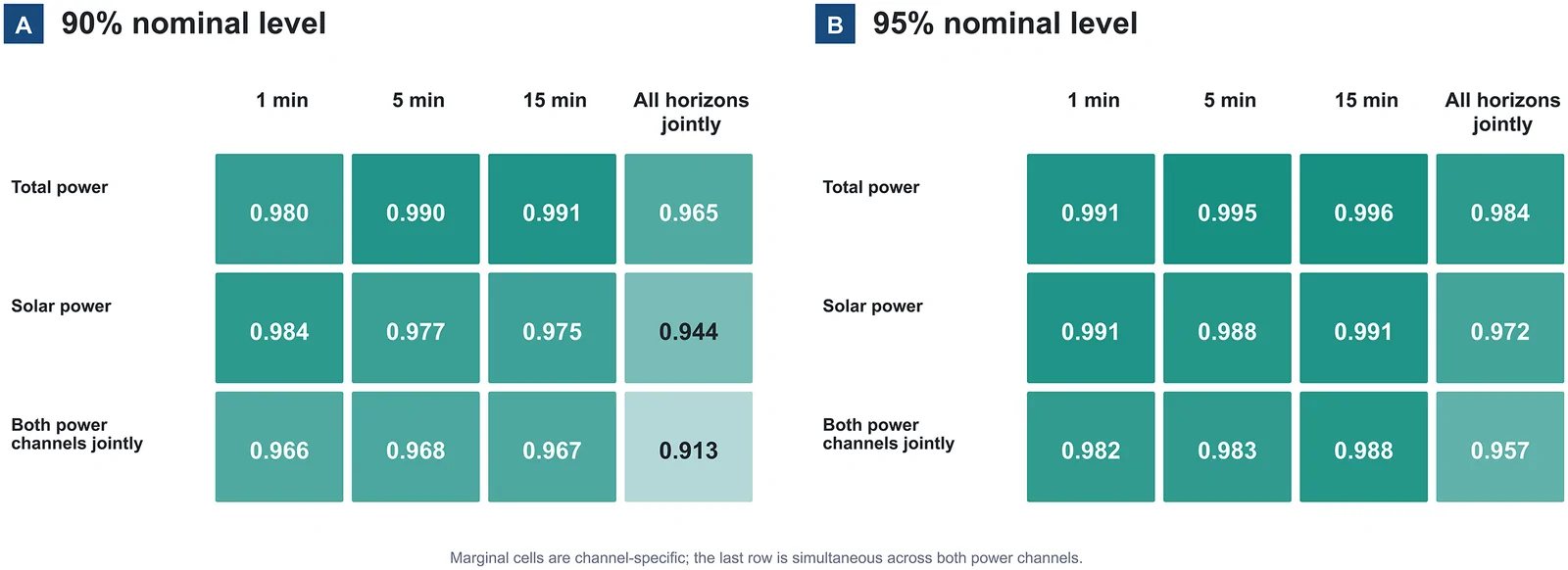
Power-channel coverage by horizon and nominal level. Cells show empirical coverage averaged across three random seeds and 25,278 July origins per seed. The final row is simultaneous across both power channels; the final column is simultaneous across all horizons.

### Paired efficiency comparisons

Paired orbit-bootstrap comparisons confirmed that the downstream selector improved power-envelope efficiency (Table 7). Relative to residual-volume selection, normalized score decreased by 1.522 (95% CI, 1.505–1.540; 34.3%) at 90% and by 1.552 (95% CI, 1.534–1.570; 30.3%) at 95%. Relative reductions were 18.1–20.1% versus ellipsoid-only task selection, 29.0–32.9% versus OAS, and 43.1–46.1% versus the direct 21-dimensional ellipsoid. All eight Holm-adjusted paired bootstrap P values were <0.001.

**Table 7:** Paired complete-orbit bootstrap comparisons of normalized power interval score.

Target	Comparator	Comparator score	Task-selected score	Absolute reduction (95% CI)	Relative reduction	Holm-adjusted P
90%	Residual-volume selection	4.443	2.921	1.522 [1.505, 1.540]	34.3%	<0.001
90%	Ellipsoid-only task selection	3.654	2.921	0.734 [0.722, 0.745]	20.1%	<0.001
90%	OAS ellipsoid	4.355	2.921	1.434 [1.417, 1.450]	32.9%	<0.001
90%	Direct 21-D ellipsoid	5.418	2.921	2.498 [2.474, 2.522]	46.1%	<0.001
95%	Residual-volume selection	5.127	3.575	1.552 [1.534, 1.570]	30.3%	<0.001
95%	Ellipsoid-only task selection	4.363	3.575	0.788 [0.773, 0.801]	18.1%	<0.001
95%	OAS ellipsoid	5.034	3.575	1.459 [1.440, 1.477]	29.0%	<0.001
95%	Direct 21-D ellipsoid	6.279	3.575	2.704 [2.677, 2.733]	43.1%	<0.001

Scores and reductions are orbit-balanced. Absolute reductions are comparator minus task-selected score. Confidence intervals use 2,000 paired complete-orbit bootstrap replicates. P values are two-sided and Holm-adjusted across eight comparator-by-target comparisons.

### Calibration-window and physical-domain sensitivity

The 56-, 112-, and 224-orbit histories and static June calibration produced closely grouped orbit-balanced coverage and normalized interval score (S1 Table; Fig 8A), confirming stable performance across the pre-specified one-week history and alternative calibration histories. Removing the non-negative intersection left primitive joint coverage unchanged for every seed and target but increased normalized power score, consistent with the fact that all observed primitive targets were non-negative. Observed ranges and zero negative fractions in all periods are documented in S2 Table.

**Fig 8:**
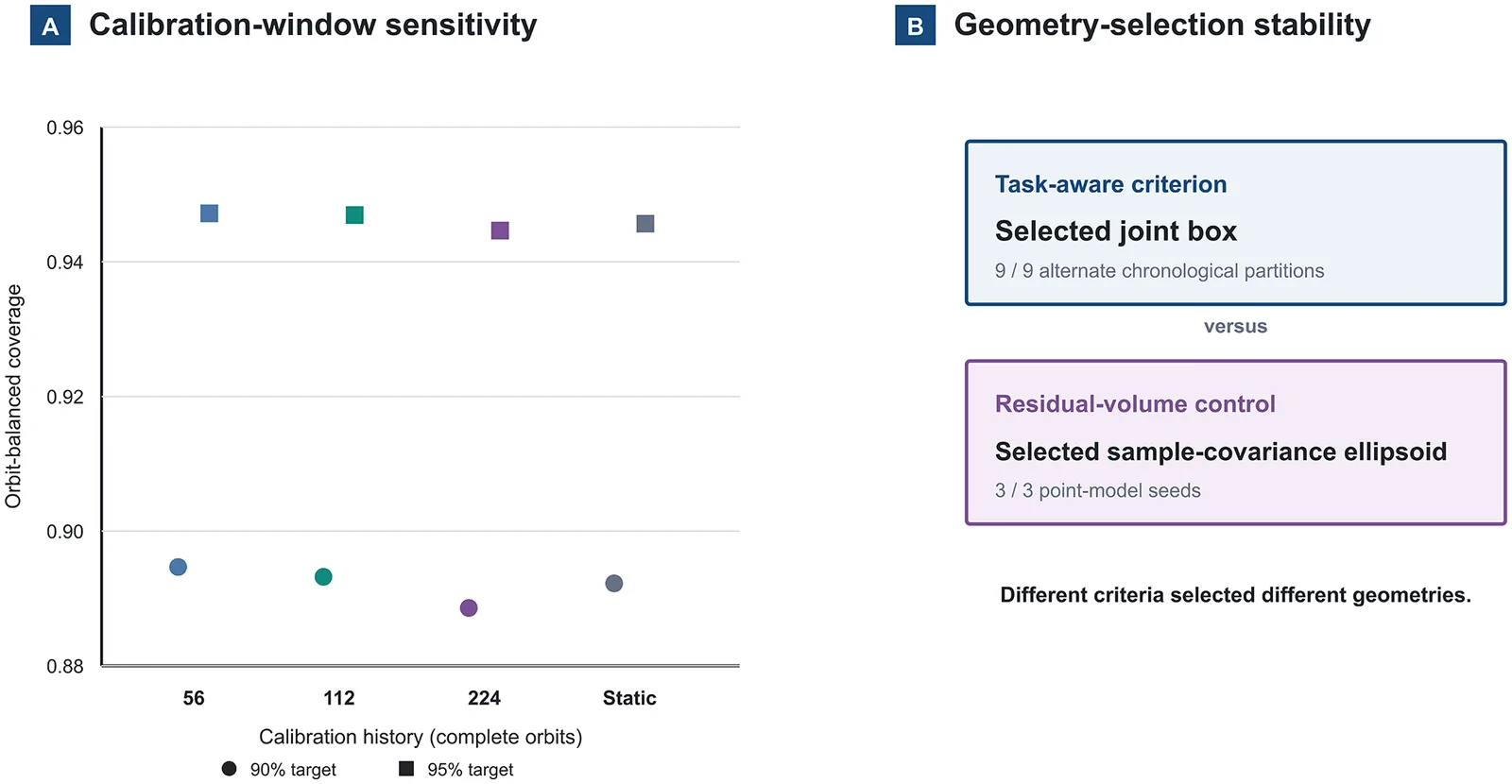
Robustness of calibration history and geometry selection. (A) Orbit-balanced primitive-state joint coverage for 56-, 112-, and 224-orbit histories and static June calibration at 90% and 95% nominal levels. (B) The task-aware criterion selected the box in nine alternate chronological partitions, whereas the residual-volume control selected the sample-covariance ellipsoid in all three random seeds.

## Discussion

The residual-volume control clarifies the selection result. A sample-covariance ellipsoid was the compact choice in standardized residual space, whereas the task-aware criterion consistently selected the box and reduced downstream power interval score by about 30–34%. The result attributes the gain to selecting residual geometry for the derived power quantity, rather than to residual-space compactness alone.

Power-channel diagnostics show that the operational quantities were covered more frequently than the full 15-coordinate primitive event. At the 90% nominal calibration level, orbit-balanced primitive joint coverage was 0.892 with a 95% complete-orbit CI of 0.883–0.899. Reporting nominal levels together with empirical estimates and orbit-based CIs directly characterizes performance under the temporally ordered evaluation protocol.

Algebraic coherence follows directly from the selected-set construction. Because synchronized currents and bus voltage define both power targets, interval arithmetic over a non-negative box returns the exact power image. The zero-gap and full-containment audit verifies implementation of this mapping. Coverage, width, interval score, geometry ranking, and calibration-history sensitivity quantify data-dependent performance.

The evaluation uses the public BUPT-1 CubeSat record from March–July 2023 and a residual-iTransformer point-centre architecture under three random seeds. This defined benchmark supports reproducible assessment of geometry selection, online calibration, and derived-power consistency. Recent thermal–electrical modeling [21] addresses complementary physics-informed point prediction; the present work addresses joint uncertainty and derived-power consistency around a fixed predictor.

Within that scope, the chronological protocol, source-pinned telemetry, complete-orbit resampling, explicit metric weighting, power-channel coverage, geometry-control experiment, and open source package make the engineering evidence inspectable and reproducible.

## Conclusions

This study developed and evaluated task-aware conformal power envelopes for multi-horizon BUPT-1 monitoring. A downstream power criterion selected a joint box in every random-seed run, whereas a residual-volume control selected the sample-covariance ellipsoid. The task-aware selector reduced normalized power interval score by 30.3–34.3% relative to that non-task-aware control, with paired 95% complete-orbit bootstrap confidence intervals excluding zero. Empirical simultaneous coverage of both power channels across all three horizons was 0.913 and 0.957 at the 90% and 95% nominal levels. Synchronized primitive-state propagation provided algebraically coherent power envelopes by construction. These results demonstrate the value of downstream geometry selection for multi-horizon spacecraft power monitoring and establish a reproducible benchmark for task-aware uncertainty-set design.

## Supporting information

S1 File Reproducibility package. Author-generated code, environment requirements, manifests, derived evidence tables, orbit-level numerical source data, and figure source data.
(ZIP)

S1 Table Calibration-window sensitivity. (CSV)

S2 Table Electrical-variable ranges, missing fractions, and negative fractions by study period. (CSV)

S3 Table Primitive coordinate-by-horizon empirical coverage. (CSV)

S4 Table Geometry-selection stability under alternate chronological orbit-block assignments. (CSV)

## References

[1] Xing R, Xu M, Zhou A, Li Q, Zhang Y, Qian F, et al. Deciphering the Enigma of Satellite Computing with COTS Devices: Measurement and Analysis. In: Proceedings of the 30th Annual International Conference on Mobile Computing and Networking, 2024. 420–35. 10.1145/3636534.3649371

[2] Kang S, Gao Y, Song Y, Zhou R, Pang J. Parameter probabilistic prediction for satellite power system based on unsupervised multi-dimensional sequence segmentation. Aerosp Sci Technol. 2024;146:108933. doi: 10.1016/j.ast.2024.108933

[3] Kang S, Yang L, Song Y, Zhou R, Pang J. Satellite Power System State Prediction Based on Online Learning With Parameter Association Rules. IEEE Trans Instrum Meas. 2023;72:1–13. doi: 10.1109/tim.2023.3291798

[4] Jiang G, Liu Y. Satellite Telemetry Parameter Prediction Based on Improved Combinatorial Machine Learning. kjkxxb. 2023;43(4):786. doi: 10.11728/cjss2023.04.2022-0057

[5] Fejjari A, Delavault A, Camilleri R, Valentino G. A review of anomaly detection in spacecraft telemetry data. Applied Sciences. 2025;15:5653. doi: 10.3390/app15105653

[6] Liu Y, Hu T, Zhang H, Wu H, Wang S, Ma L, et al. iTransformer: inverted Transformers are effective for time series forecasting. In: 2024.

[7] Lin S, Lin W, Hu X, Wu W, Mo R, Zhong H. CycleNet: Enhancing Time Series Forecasting through Modeling Periodic Patterns. In: Advances in Neural Information Processing Systems 37, 2024. 106315–45. 10.52202/079017-3373

[8] Wang S, Wu H, Shi X, Hu T, Luo H, Ma L. TimeMixer: decomposable multiscale mixing for time series forecasting. In: 2024.

[9] Angelopoulos AN, Bates S. Conformal Prediction: A Gentle Introduction. Foundations and Trends® in Machine Learning. 2023;16(4):494–591. doi: 10.1561/2200000101

[10] Portela A, Banga JR, Matabuena M. Conformal prediction for uncertainty quantification in dynamic biological systems. PLoS Comput Biol. 2025;21(5):e1013098. doi: 10.1371/journal.pcbi.1013098 40354480 PMC12091895

[11] Gibbs I, Candès EJ. Conformal inference for online prediction with arbitrary distribution shifts. J Mach Learn Res. 2024;25(162):1–36.

[12] Oliveira RI, Orenstein P, Ramos T, Romano JV. Split conformal prediction and non-exchangeable data. J Mach Learn Res. 2024;25(225):1–38.

[13] Xu C, Jiang H, Xie Y. Conformal prediction for multi-dimensional time series by ellipsoidal sets. Proceedings of Machine Learning Research. 2024;235:55076–99.

[14] Galvão Lopes A, Goubault E, Putot S, Pautet L. ConForME: multi-horizon conditional conformal time series forecasting. Proceedings of Machine Learning Research. 2024;230:345–65.

[15] Schlembach F, Smirnov E, Koprinska I, Winands MHM. Conformal multistep-ahead multivariate time-series forecasting. Mach Learn. 2025;114:165. doi: 10.1007/s10994-024-06722-9

[16] English E, Wong-Toi E, Fontana M, Mandt S, Smyth P, Lippert C. JANET: Joint Adaptive predictioN-region Estimation for Time-series. Mach Learn. 2025;114:177. doi: 10.1007/s10994-025-06812-2

[17] Cleaveland M, Lee I, Pappas GJ, Lindemann L. Conformal Prediction Regions for Time Series Using Linear Complementarity Programming. AAAI. 2024;38(19):20984–92. doi: 10.1609/aaai.v38i19.30089

[18] Tumu R, Cleaveland M, Mangharam R, Pappas G, Lindemann L. Multi-modal conformal prediction regions by optimizing convex shape templates. Proceedings of Machine Learning Research. 2024;242:1343–56.

[19] Chen Y, Wiesel A, Eldar YC, Hero AO. Shrinkage Algorithms for MMSE Covariance Estimation. IEEE Trans Signal Process. 2010;58(10):5016–29. doi: 10.1109/tsp.2010.2053029

[20] Gneiting T, Raftery AE. Strictly proper scoring rules, prediction, and estimation. J Am Stat Assoc. 2007;102:359–78. doi: 10.1198/016214506000001437

[21] Xie J, Li YZ. Telemetry-driven physics-informed prediction for on-orbit thermal–electrical performance of solar panels in satellite power system. Aerosp Sci Technol. 2026;177:112268. doi: 10.1016/j.ast.2026.112268

