# Supplementary material for: Task-aware conformal power envelopes for multi-horizon spacecraft monitoring
Source: PLoS One. 2026 Oct 13;21(10):e0360910. doi: 10.1371/journal.pone.0360910 (PMC13660891; doi:10.1371/journal.pone.0360910)
Supplement: Supplementary file 1 [file pone.0360910.s007.docx]

**Response to the Academic Editor and Reviewers**

Manuscript ID: PONE-D-26-42395

Title: Task-aware conformal power envelopes for multi-horizon spacecraft monitoring

Dear Dr. Sun and Reviewers,

Thank you for the constructive assessment. We performed additional control experiments, coverage analyses, paired complete-orbit bootstrap inference, physical-data audits, calibration-window sensitivity analyses, and geometry-stability tests. We also corrected two method-description errors: the analysis uses bus voltage—not battery voltage—in the solar-power equation, and preprocessing uses arithmetic-mean—not median—10-s aggregation. These corrections align the manuscript with the unchanged code and numerical evidence. Substantive revisions are highlighted in yellow in the marked manuscript.

# Academic Editor and journal requirements

**Requirement 1**

*Ensure PLOS ONE style requirements, including file naming, are met.*

**Response:** We reformatted the manuscript with continuous line numbers, page numbers, consistent double spacing, standardized headings and indentation, and separate publication-quality figure files. Submission files now use short manuscript-ID-based names.

**Requirement 2**

*Author-generated code underpinning the findings must be available without restriction upon publication.*

**Response:** S1 File now contains all author-generated preprocessing, training, evaluation, bootstrap, table, and figure scripts; environment requirements; source and split manifests; derived source data; and an MIT license. The public telemetry repository and pinned commit are specified in the manuscript and package.

**Requirement 3**

*Funding Information and Financial Disclosure do not match.*

**Response:** The funding statement has been standardized in the manuscript, cover letter, and response: Ministry of Industry and Information Technology of the People’s Republic of China, Grant 5120230637; and Ningbo Commonweal Programme, Zhejiang Province, Grant 2025S061. The no-role statement is also included.

**Requirement 4**

*Refer to Table 1 in the manuscript text.*

**Response:** The Data source, preprocessing, and electrical variables subsection now explicitly introduces Table 1 before the table.

**Requirement 5**

*Evaluate reviewer-suggested publications for relevance.*

**Response:** We evaluated the suggested works and added the directly relevant 2023 satellite telemetry forecasting study, the 2023 satellite power online state-prediction study, and the 2025 spacecraft telemetry review. The Introduction now distinguishes point/interval telemetry prediction, adaptive satellite power-state prediction, and the present downstream conformal geometry problem. We did not add citations solely because they were suggested.

# Reviewer 1

**Comment 1**

*Strengthen related work and positioning in spacecraft telemetry forecasting, interval prediction, power-system state prediction, and physics-informed modeling.*

**Response:** The Introduction now includes verified spacecraft-specific studies by Jiang and Liu (2023), Kang et al. (2023, 2024), Fejjari et al. (2025), and Xie and Li (2026), and explains the boundary between point/interval forecasting, adaptive state prediction, anomaly monitoring, physics-informed point prediction, and task-aware conformal geometry selection.

**Comment 2**

*Distinguish nominal, empirical, and theoretical coverage under temporal dependence.*

**Response:** We now call 90% and 95% nominal calibration targets, report July empirical coverage with 95% complete-orbit bootstrap CIs, and explicitly state that the dependent completed-orbit protocol is not presented as an exact exchangeable split-conformal guarantee. The Discussion separately interprets the 0.892 empirical estimate and its 0.883–0.899 CI.

**Comment 3**

*Add a stronger non-ellipsoidal baseline or geometry selected without the downstream power criterion.*

**Response:** We added a residual-volume geometry selector that does not use power. It selected the sample-covariance ellipsoid in all three seeds, whereas the task-aware criterion selected the box. The task-aware method reduced normalized power interval score by 34.3% at 90% and 30.3% at 95% relative to this control, with paired 95% CIs reported in Table 7 and Fig 5.

**Comment 4**

*Justify the 112-orbit calibration window and add sensitivity analysis.*

**Response:** The revised Methods state that 112 orbits approximate a pre-specified seven-day operational memory. We added 56-, 112-, and 224-orbit histories and static June calibration. Results are reported in S1 Table and Fig 8A.

**Comment 5**

*Clarify duplicate removal, discontinuities, missing data, 13 channels, scaling, and validation period.*

**Response:** The revised preprocessing subsection now specifies 10-s arithmetic means, the eight-raw-sample threshold, duplicate handling, the exact 10-s discontinuity rule, 35 continuous segments, zero audited missing values, all 13 input channels, training-only scaling, and June 1–30 point-model early stopping. Table 1 also clarifies the June role.

**Comment 6**

*Describe algebraic consistency as by construction rather than purely empirical.*

**Response:** We now state that exact interval-image equality for the selected non-negative box follows from interval arithmetic. The zero-gap audit is described as an implementation check; coverage, width, and interval score remain empirical results.

**Comment 7**

*Moderate generalization claims or provide additional operational-condition results.*

**Response:** We limit conclusions to one BUPT-1 CubeSat, the March–July 2023 season, and one point-centre architecture under three random seeds. We added coverage by channel and horizon, calibration-history sensitivity, and nine alternate geometry-development partitions, but do not claim cross-spacecraft generalization.

# Reviewer 2

**Comment 1**

*Report power coverage by channel, horizon, joint event, and primitive variable.*

**Response:** New Table 6 and Fig 7 report total-power, solar-power, and both-channel simultaneous coverage at 1, 5, and 15 min and jointly across all horizons. S3 Table reports every primitive coordinate-by-horizon coverage, and orbit-level source data are included in S1 File.

**Comment 2**

*Complete metric definitions and statistical inference.*

**Response:** The revised Methods define normalization scales, equal 1/6 channel-horizon weights, sample- and orbit-balanced aggregation, dimensionless units, and the interval score. New Table 7 reports absolute paired reductions, relative reductions, 95% complete-orbit bootstrap CIs, and Holm-adjusted P values across eight planned comparator-by-target tests.

**Comment 3**

*Provide the physical basis, sign convention, units, ranges, synchronization, and sensitivity without nonnegative truncation.*

**Response:** S2 Table reports period-specific units, ranges, missing fractions, and negative fractions. All five primitive targets were non-negative in every period. The manuscript now confirms that both power equations use synchronized same-bin bus voltage. A no-intersection sensitivity analysis preserved joint coverage but increased interval score; full numerical results are in S1 File. The displayed solar-power voltage symbol was corrected from battery voltage to bus voltage to match the code and data.

**Comment 4**

*Clarify scope, random-seed wording, geometry stability, and the width criterion.*

**Response:** We replaced ‘three independently trained point models’ with ‘three random-seed runs.’ We limit claims to the single-satellite case, added nine alternate orbit-block assignments, and explain that normalized width is the selection-only sharpness surrogate while interval score is used for final evaluation.

**Comment 5**

*Align formatting, define abbreviations, and standardize captions and statistics.*

**Response:** The revised manuscript adds continuous line and page numbering, double spacing, consistent heading levels and decimal precision, and first-use definitions for RMSE, MPPT, OAS, and low-Earth orbit where used. Captions identify sample sizes and whether uncertainty bars are seed standard deviations or 95% complete-orbit bootstrap CIs.

**Comment 6**

*Improve stand-alone figures and provide transparent source data.*

**Response:** Fig 1 was redrawn to show the residual-volume control and structural power map. Fig 5 now uses paired complete-orbit bootstrap CIs. New Fig 7 reports coverage by power channel and horizon, and new Fig 8 reports calibration-window and geometry-selection stability. Figures use a colorblind-friendly palette and are supplied as 300-dpi TIFF files; underlying CSV data and SVG-generation code are in S1 File.

We believe the new controls and direct operational coverage analyses substantially strengthen the manuscript. We appreciate the reviewers’ guidance and have checked the numerical claims against the frozen July evaluation outputs.

Sincerely,
Nan Lu, on behalf of all authors
