# Supplementary material for: Task-aware conformal power envelopes for multi-horizon spacecraft monitoring
Source: PLoS One. 2026 Oct 13;21(10):e0360910. doi: 10.1371/journal.pone.0360910 (PMC13660891; doi:10.1371/journal.pone.0360910)
Supplement: Peer review history

# Peer review: Decision Letter 0

**DOI:** 10.1371/journal.pone.0360910.r001

**Authors:** Guangyu Sun

17 Sep 2026

Dear Dr. Lu,

Thank you for submitting your manuscript to PLOS One. After careful consideration, we feel that it has merit but does not fully meet PLOS One’s publication criteria as it currently stands. Therefore, we invite you to submit a revised version of the manuscript that addresses the points raised during the review process.

Please submit your revised manuscript by Nov 16 2026 11:59PM. If you will need more time than this to complete your revisions, please reply to this message or contact the journal office at plosone@plos.org. When you're ready to submit your revision, log on to https://www.editorialmanager.com/pone/ and select the 'Submissions Needing Revision' folder to locate your manuscript file.

- A letter that responds to each point raised by the academic editor and reviewer(s). You should upload this letter as a separate file labeled 'Response to Reviewers'.
- A marked-up copy of your manuscript that highlights changes made to the original version. You should upload this as a separate file labeled 'Revised Manuscript with Track Changes'.
- An unmarked version of your revised paper without tracked changes. You should upload this as a separate file labeled 'Manuscript'.

If you would like to make changes to your financial disclosure, please include your updated statement in your cover letter. Guidelines for resubmitting your figure files are available below the reviewer comments at the end of this letter.

If applicable, we recommend that you deposit your laboratory protocols in protocols.io to enhance the reproducibility of your results. Protocols.io assigns your protocol its own identifier (DOI) so that it can be cited independently in the future. For instructions see: https://journals.plos.org/plosone/s/submission-guidelines#loc-laboratory-protocols. Additionally, PLOS One offers an option for publishing peer-reviewed Lab Protocol articles, which describe protocols hosted on protocols.io. Read more information on sharing protocols at https://plos.org/protocols?utm_medium=editorial-email&utm_source=authorletters&utm_campaign=protocols.

As the corresponding author, your ORCID iD is verified in the submission system and will appear in the published article. PLOS supports the use of ORCID, and we encourage all coauthors to register for an ORCID iD and use it as well. Please encourage your coauthors to verify their ORCID iD within the submission system before final acceptance, as unverified ORCID iDs will not appear in the published article. *Only*  the individual author can complete the verification step; PLOS staff *cannot*  verify ORCID iDs on behalf of authors.

We look forward to receiving your revised manuscript.

Kind regards,

Guangyu Sun, Ph.D

Academic Editor

PLOS One

Journal Requirements:

When submitting your revision, we need you to address these additional requirements.

1. Please ensure that your manuscript meets PLOS ONE's style requirements, including those for file naming. The PLOS ONE style templates can be found at

https://journals.plos.org/plosone/s/file?id=wjVg/PLOSOne_formatting_sample_main_body.pdf and

https://journals.plos.org/plosone/s/file?id=ba62/PLOSOne_formatting_sample_title_authors_affiliations.pdf

2. Please note that PLOS One has specific guidelines on code sharing for submissions in which author-generated code underpins the findings in the manuscript. In these cases, we expect all author-generated code to be made available without restrictions upon publication of the work. Please review our guidelines at https://journals.plos.org/plosone/s/materials-and-software-sharing#loc-sharing-code and ensure that your code is shared in a way that follows best practice and facilitates reproducibility and reuse.

3. We note that the grant information you provided in the ‘Funding Information’ and ‘Financial Disclosure’ sections do not match.

When you resubmit, please ensure that you provide the correct grant numbers for the awards you received for your study in the ‘Funding Information’ section.

4. We note you have included a table to which you do not refer in the text of your manuscript. Please ensure that you refer to Table 1 in your text; if accepted, production will need this reference to link the reader to the Table.

5. If the reviewer comments include a recommendation to cite specific previously published works, please review and evaluate these publications to determine whether they are relevant and should be cited. There is no requirement to cite these works unless the editor has indicated otherwise.

[Note: HTML markup is below. Please do not edit.]

Reviewers' comments:

Reviewer's Responses to Questions

**Comments to the Author**

1. Is the manuscript technically sound, and do the data support the conclusions?

Reviewer #1: Yes

Reviewer #2: Yes

**********

2. Has the statistical analysis been performed appropriately and rigorously?

Reviewer #1: Yes

Reviewer #2: Yes

**********

3. Have the authors made all data underlying the findings in their manuscript fully available?

The PLOS Data policy

Reviewer #1: Yes

Reviewer #2: Yes

**********

4. Is the manuscript presented in an intelligible fashion and written in standard English?

Reviewer #1: Yes

Reviewer #2: No

**********

Reviewer #1: The manuscript titled Task-aware conformal power envelopes for multi-horizon spacecraft monitoring presents a technically interesting framework for task-aware conformal uncertainty quantification in spacecraft power monitoring. The chronological data split, completed-orbit updating, and algebraically coherent propagation from primitive states to derived power are strengths. However, several issues should be addressed before publication.

1.Related work and positioning. The Introduction should better position the study relative to prior spacecraft telemetry forecasting, prediction-interval, power-system state-prediction, and physics-informed modeling work. Relevant examples include:

(1)Luan et al., Prediction Interval on Spacecraft Telemetry Data Based on Modified Block Bootstrap Method, 2010.

(2)Jiang and Liu, Satellite Telemetry Parameter Prediction Based on Improved Combinatorial Machine Learning, 2023.

(3)Kang et al., Satellite Power System State Prediction Based on Online Learning With Parameter Association Rules, IEEE TIM, 2023.

(4)Fejjari et al., A Review of Anomaly Detection in Spacecraft Telemetry Data, 2025.

(5)Li et al., Spin-Modulated Thermoelastic Response of a Flexible Spacecraft Appendage Under Attitude-Dependent Solar Radiation, Aerospace, 2026.

These are examples rather than required citations; the main need is clearer positioning of the present contribution.

2.The interpretation of conformal coverage should be clarified. Because spacecraft telemetry is temporally dependent, the manuscript should distinguish between nominal coverage, empirical coverage, and theoretical coverage guarantees. This is particularly relevant because the reported 90% orbit-balanced coverage is 0.892.

3.Stronger baselines are needed. The current comparison mainly includes a joint box and several ellipsoidal regions. Since the joint box is selected in all cases, it would be useful to include at least one stronger non-ellipsoidal baseline or a geometry selected without the downstream power criterion, to better isolate the contribution of the task-aware selection strategy.

4.The choice of the 112-orbit calibration window should be justified. The manuscript should explain how this value was determined and whether it was fixed before testing. A brief sensitivity analysis using different window lengths would strengthen the robustness of the results.

5.More implementation details are required for reproducibility. The manuscript should clarify duplicate removal, discontinuity detection, missing-data handling, the 13 input channels, scaling procedures, and the exact validation period used for hyperparameter selection.

6.The algebraic-consistency results should be described more carefully. For the selected non-negative box, zero algebraic gap and full containment are largely guaranteed by interval arithmetic rather than being purely empirical findings. The manuscript should explicitly distinguish these by-construction properties from data-driven performance results.

7.The generalization claims should be moderated. The evaluation is based on one BUPT-1 CubeSat and a limited July 2023 period. Additional stratified results under different operational conditions would be helpful; otherwise, the limitation of single-spacecraft validation should be clearly stated.

Overall recommendation: Major Revision. The manuscript has a promising methodological contribution, but the literature review, statistical interpretation, baseline comparisons, reproducibility, and generalization analysis should be strengthened.

Reviewer #2: The manuscript proposes a task-aware conformal framework for multi-horizon spacecraft power monitoring. A residual iTransformer provides point centers, box and ellipsoid sets are fitted, selected, and calibrated on chronologically separated complete-orbit blocks, calibration is updated by orbit in July, and interval arithmetic produces total- and solar-power envelopes. The problem is well motivated, and the algebraic propagation strategy is valuable. However, online calibration, coverage reporting, baseline fairness, physical assumptions, data processing, and reproducibility require further clarification. Major revision is recommended.

1. The coverage target is not fully aligned with the operational quantities of interest.

The current procedure covers a 15-dimensional set comprising five primitive variables at three horizons, whereas the application outcomes are total and solar power. Please report marginal and simultaneous coverage for total power, solar power, their joint event, each horizon, and each primitive variable. At minimum, the main text should report coverage directly relevant to the two power channels; additional diagnostics by orbit, day, or variable can be provided as supplementary material.

2. Metric definitions and statistical uncertainty need to be completed.

Please define precisely how normalized width and interval score are computed, including the weights assigned to variables, horizons, and power channels; the normalization scales; the aggregation method; and the final units. Standard deviations across seeds in Table 5 are not substitutes for inferential confidence intervals. Major percentage improvements should be accompanied by 95% paired orbit-block bootstrap confidence intervals and absolute differences, with a statement on whether multiplicity was addressed across seeds, candidates, and target levels.

3. The physical basis for the nonnegativity assumption and power equations is insufficient.

Please provide the measurement definition, sign convention, units, and plausible range for each electrical variable, and report minimum values and negative fractions in the training, validation, and test periods. If currents can change sign, explain how interval arithmetic handles this and provide sensitivity analyses without truncating the nonnegative orthant. Please also confirm that the currents and voltage in Psol=(Imppt1+Imppt2)Ubat use the same voltage basis and synchronized timestamps; otherwise, the claimed physical consistency may exist only at the level of the equations.

4. External validity and scope should be clarified.

The phrase “three independently trained point models” should be changed to “three random seeds” to avoid implying three different model classes. The current evidence comes from one satellite, one season, and a test period of approximately 24 days. If the authors retain claims that the method is compatible with any point predictor or generalizable to spacecraft monitoring, additional validation on another satellite, season, or point predictor is recommended. If such validation is not feasible, the conclusions should be explicitly limited to this single-satellite case study. The authors could also report geometry-selection stability under alternative orbit-block assignments and justify width as a reasonable surrogate for interval score.

5. Align the manuscript with submission requirements.

Please add continuous line numbers and page numbers and standardize line spacing, heading levels, indentation, decimal precision, and mathematical notation. Define abbreviations such as RMSE, MPPT, OAS, and LEO at first use, and include units, sample sizes, and definitions of reported statistics in tables and figure captions. Terminology should be consistent across the Abstract, main text, and figure captions.

6. Improve the stand-alone readability of figures and tables and the transparency of their underlying data.

Please ensure that Figs. 1-6 meet journal requirements for resolution, file format, legends, and axis labels; use colorblind-friendly palettes; identify error bars explicitly as standard deviations or confidence intervals; and report sample sizes for each panel. Consider adding plots of coverage over time, stability of candidate-geometry rankings, and errors for the representative orbit. Source data for the figures should be provided as supplementary material where feasible.

**********

what does this mean?). If published, this will include your full peer review and any attached files.

If you choose “no”, your identity will remain anonymous but your review may still be made public.

**Do you want your identity to be public for this peer review?**  For information about this choice, including consent withdrawal, please see our Privacy Policy

Reviewer #1: No

Reviewer #2: No

**********

[NOTE: If reviewer comments were submitted as an attachment file, they will be attached to this email and accessible via the submission site. Please log into your account, locate the manuscript record, and check for the action link "View Attachments". If this link does not appear, there are no attachment files.]

To ensure your figures meet our technical requirements, please review our figure guidelines: https://journals.plos.org/plosone/s/figures

You may also use PLOS’s free figure tool, NAAS, to help you prepare publication quality figures: https://journals.plos.org/plosone/s/figures#loc-tools-for-figure-preparation.

NAAS will assess whether your figures meet our technical requirements by comparing each figure against our figure specifications.

# Author comment: Author response to Decision Letter 1

**DOI:** 10.1371/journal.pone.0360910.r002

21 Sep 2026

Response to the Academic Editor and Reviewers

Manuscript ID: PONE-D-26-42395

Title: Task-aware conformal power envelopes for multi-horizon spacecraft monitoring

Dear Dr. Sun and Reviewers,

Thank you for the constructive assessment. We performed additional control experiments, coverage analyses, paired complete-orbit bootstrap inference, physical-data audits, calibration-window sensitivity analyses, and geometry-stability tests. We also corrected two method-description errors: the analysis uses bus voltage—not battery voltage—in the solar-power equation, and preprocessing uses arithmetic-mean—not median—10-s aggregation. These corrections align the manuscript with the unchanged code and numerical evidence. Substantive revisions are highlighted in yellow in the marked manuscript.

Academic Editor and journal requirements

Requirement 1

Ensure PLOS ONE style requirements, including file naming, are met.

Response: We reformatted the manuscript with continuous line numbers, page numbers, consistent double spacing, standardized headings and indentation, and separate publication-quality figure files. Submission files now use short manuscript-ID-based names.

Requirement 2

Author-generated code underpinning the findings must be available without restriction upon publication.

Response: S1 File now contains all author-generated preprocessing, training, evaluation, bootstrap, table, and figure scripts; environment requirements; source and split manifests; derived source data; and an MIT license. The public telemetry repository and pinned commit are specified in the manuscript and package.

Requirement 3

Funding Information and Financial Disclosure do not match.

Response: The funding statement has been standardized in the manuscript, cover letter, and response: Ministry of Industry and Information Technology of the People’s Republic of China, Grant 5120230637; and Ningbo Commonweal Programme, Zhejiang Province, Grant 2025S061. The no-role statement is also included.

Requirement 4

Refer to Table 1 in the manuscript text.

Response: The Data source, preprocessing, and electrical variables subsection now explicitly introduces Table 1 before the table.

Requirement 5

Evaluate reviewer-suggested publications for relevance.

Response: We evaluated the suggested works and added the directly relevant 2023 satellite telemetry forecasting study, the 2023 satellite power online state-prediction study, and the 2025 spacecraft telemetry review. The Introduction now distinguishes point/interval telemetry prediction, adaptive satellite power-state prediction, and the present downstream conformal geometry problem. We did not add citations solely because they were suggested.

Reviewer 1

Comment 1

Strengthen related work and positioning in spacecraft telemetry forecasting, interval prediction, power-system state prediction, and physics-informed modeling.

Response: The Introduction now includes verified spacecraft-specific studies by Jiang and Liu (2023), Kang et al. (2023, 2024), Fejjari et al. (2025), and Xie and Li (2026), and explains the boundary between point/interval forecasting, adaptive state prediction, anomaly monitoring, physics-informed point prediction, and task-aware conformal geometry selection.

Comment 2

Distinguish nominal, empirical, and theoretical coverage under temporal dependence.

Response: We now call 90% and 95% nominal calibration targets, report July empirical coverage with 95% complete-orbit bootstrap CIs, and explicitly state that the dependent completed-orbit protocol is not presented as an exact exchangeable split-conformal guarantee. The Discussion separately interprets the 0.892 empirical estimate and its 0.883–0.899 CI.

Comment 3

Add a stronger non-ellipsoidal baseline or geometry selected without the downstream power criterion.

Response: We added a residual-volume geometry selector that does not use power. It selected the sample-covariance ellipsoid in all three seeds, whereas the task-aware criterion selected the box. The task-aware method reduced normalized power interval score by 34.3% at 90% and 30.3% at 95% relative to this control, with paired 95% CIs reported in Table 7 and Fig 5.

Comment 4

Justify the 112-orbit calibration window and add sensitivity analysis.

Response: The revised Methods state that 112 orbits approximate a pre-specified seven-day operational memory. We added 56-, 112-, and 224-orbit histories and static June calibration. Results are reported in S1 Table and Fig 8A.

Comment 5

Clarify duplicate removal, discontinuities, missing data, 13 channels, scaling, and validation period.

Response: The revised preprocessing subsection now specifies 10-s arithmetic means, the eight-raw-sample threshold, duplicate handling, the exact 10-s discontinuity rule, 35 continuous segments, zero audited missing values, all 13 input channels, training-only scaling, and June 1–30 point-model early stopping. Table 1 also clarifies the June role.

Comment 6

Describe algebraic consistency as by construction rather than purely empirical.

Response: We now state that exact interval-image equality for the selected non-negative box follows from interval arithmetic. The zero-gap audit is described as an implementation check; coverage, width, and interval score remain empirical results.

Comment 7

Moderate generalization claims or provide additional operational-condition results.

Response: We limit conclusions to one BUPT-1 CubeSat, the March–July 2023 season, and one point-centre architecture under three random seeds. We added coverage by channel and horizon, calibration-history sensitivity, and nine alternate geometry-development partitions, but do not claim cross-spacecraft generalization.

Reviewer 2

Comment 1

Report power coverage by channel, horizon, joint event, and primitive variable.

Response: New Table 6 and Fig 7 report total-power, solar-power, and both-channel simultaneous coverage at 1, 5, and 15 min and jointly across all horizons. S3 Table reports every primitive coordinate-by-horizon coverage, and orbit-level source data are included in S1 File.

Comment 2

Complete metric definitions and statistical inference.

Response: The revised Methods define normalization scales, equal 1/6 channel-horizon weights, sample- and orbit-balanced aggregation, dimensionless units, and the interval score. New Table 7 reports absolute paired reductions, relative reductions, 95% complete-orbit bootstrap CIs, and Holm-adjusted P values across eight planned comparator-by-target tests.

Comment 3

Provide the physical basis, sign convention, units, ranges, synchronization, and sensitivity without nonnegative truncation.

Response: S2 Table reports period-specific units, ranges, missing fractions, and negative fractions. All five primitive targets were non-negative in every period. The manuscript now confirms that both power equations use synchronized same-bin bus voltage. A no-intersection sensitivity analysis preserved joint coverage but increased interval score; full numerical results are in S1 File. The displayed solar-power voltage symbol was corrected from battery voltage to bus voltage to match the code and data.

Comment 4

Clarify scope, random-seed wording, geometry stability, and the width criterion.

Response: We replaced ‘three independently trained point models’ with ‘three random-seed runs.’ We limit claims to the single-satellite case, added nine alternate orbit-block assignments, and explain that normalized width is the selection-only sharpness surrogate while interval score is used for final evaluation.

Comment 5

Align formatting, define abbreviations, and standardize captions and statistics.

Response: The revised manuscript adds continuous line and page numbering, double spacing, consistent heading levels and decimal precision, and first-use definitions for RMSE, MPPT, OAS, and low-Earth orbit where used. Captions identify sample sizes and whether uncertainty bars are seed standard deviations or 95% complete-orbit bootstrap CIs.

Comment 6

Improve stand-alone figures and provide transparent source data.

Response: Fig 1 was redrawn to show the residual-volume control and structural power map. Fig 5 now uses paired complete-orbit bootstrap CIs. New Fig 7 reports coverage by power channel and horizon, and new Fig 8 reports calibration-window and geometry-selection stability. Figures use a colorblind-friendly palette and are supplied as 300-dpi TIFF files; underlying CSV data and SVG-generation code are in S1 File.

We believe the new controls and direct operational coverage analyses substantially strengthen the manuscript. We appreciate the reviewers’ guidance and have checked the numerical claims against the frozen July evaluation outputs.

Sincerely,

Nan Lu, on behalf of all authors

Attachment Submitted filename: PONE-D-26-42395_Response_to_Reviewers.docx

# Peer review: Decision Letter 1

**DOI:** 10.1371/journal.pone.0360910.r003

**Authors:** Guangyu Sun

5 Oct 2026

Task-aware conformal power envelopes for multi-horizon spacecraft monitoring

PONE-D-26-42395R1

Dear Dr. Lu,

We’re pleased to inform you that your manuscript has been judged scientifically suitable for publication and will be formally accepted for publication once it meets all outstanding technical requirements.

Within one week, you’ll receive an e-mail detailing the required amendments. When these have been addressed, you’ll receive a formal acceptance letter and your manuscript will be scheduled for publication.

An invoice will be generated when your article is formally accepted. Please note, if your institution has a publishing partnership with PLOS and your article meets the relevant criteria, all or part of your publication costs will be covered. Please make sure your user information is up-to-date by logging into Editorial Manager at Editorial Manager® and clicking the ‘Update My Information' link at the top of the page. For questions related to billing, please contact billing support.

If your institution or institutions have a press office, please notify them about your upcoming paper to help maximize its impact. If they’ll be preparing press materials, please inform our press team as soon as possible -- no later than 48 hours after receiving the formal acceptance. Your manuscript will remain under strict press embargo until 2 pm Eastern Time on the date of publication. For more information, please contact onepress@plos.org.

Kind regards,

Guangyu Sun, Ph.D

Academic Editor

PLOS One

Additional Editor Comments (optional):

Reviewers' comments:

Reviewer's Responses to Questions

**Comments to the Author**

Reviewer #1: All comments have been addressed

Reviewer #2: All comments have been addressed

**********

2. Is the manuscript technically sound, and do the data support the conclusions?

Reviewer #1: Yes

Reviewer #2: Yes

**********

3. Has the statistical analysis been performed appropriately and rigorously?

Reviewer #1: Yes

Reviewer #2: Yes

**********

4. Have the authors made all data underlying the findings in their manuscript fully available?

The PLOS Data policy

Reviewer #1: Yes

Reviewer #2: Yes

**********

5. Is the manuscript presented in an intelligible fashion and written in standard English?

Reviewer #1: Yes

Reviewer #2: Yes

**********

Reviewer #1: The authors have carefully addressed all the comments and concerns raised in the previous round of review. The manuscript has been revised accordingly, and the responses to the reviewers’ comments are satisfactory. I believe that the revised manuscript has been substantially improved and is now suitable for publication.

Reviewer #2: The authors have satisfactorily addressed all of my previous comments, and the revised manuscript is substantially improved. I have no further substantive concerns and recommend acceptance.

**********

what does this mean?). If published, this will include your full peer review and any attached files.

If you choose “no”, your identity will remain anonymous but your review may still be made public.

**Do you want your identity to be public for this peer review?**  For information about this choice, including consent withdrawal, please see our Privacy Policy

Reviewer #1: No

Reviewer #2: No

**********

# Editor report: Acceptance letter

**DOI:** 10.1371/journal.pone.0360910.r004

**Authors:** Guangyu Sun

PONE-D-26-42395R1

PLOS One

Dear Dr. Lu,

I'm pleased to inform you that your manuscript has been deemed suitable for publication in PLOS One. Congratulations! Your manuscript is now being handed over to our production team.

At this stage, our production department will prepare your paper for publication. This includes ensuring the following:

* All references, tables, and figures are properly cited

* All relevant supporting information is included in the manuscript submission,

* There are no issues that prevent the paper from being properly typeset

You will receive further instructions from the production team, including instructions on how to review your proof when it is ready. Please keep in mind that we are working through a large volume of accepted articles, so please give us a few days to review your paper and let you know the next and final steps.

Lastly, if your institution or institutions have a press office, please let them know about your upcoming paper now to help maximize its impact. If they'll be preparing press materials, please inform our press team within the next 48 hours. Your manuscript will remain under strict press embargo until 2 pm Eastern Time on the date of publication. For more information, please contact onepress@plos.org.

You will receive an invoice from PLOS for your publication fee after your manuscript has reached the completed accept phase. If you receive an email requesting payment before acceptance or for any other service, this may be a phishing scheme. Learn how to identify phishing emails and protect your accounts at https://explore.plos.org/phishing.

If we can help with anything else, please email us at customercare@plos.org.

Thank you for submitting your work to PLOS One and supporting open access.

Kind regards,

PLOS One Editorial Office Staff

on behalf of

Dr. Guangyu Sun

Academic Editor

PLOS One
